# Suprachoroidal injection of triamcinolone acetonide plus intravitreal bevacizumab in diabetic macular edema: a randomized pilot trial

**DOI:** 10.1186/s12886-023-02790-y

**Published:** 2023-01-27

**Authors:** Farhad Fazel, Mohammad Malekahmadi, Awat Feizi, Behrooz Oliya, Mehdi Tavakoli, Mohammadreza Fazel

**Affiliations:** 1 Isfahan Eye Research Center, Department of Ophthalmology, Isfahan University of Medical Sciences, Isfahan, Iran; 2 Cardiac Rehabilitation Research Center, Cardiovascular Research Institute, Isfahan University of Medical Sciences, Isfahan, Iran

**Keywords:** Suprachoroidal, Triamcinolone acetonide, Bevacizumab, Center-involving, Diabetic macular edema

## Abstract

**Background:**

To investigate the efficacy of injecting suprachoroidal triamcinolone acetonide (SCTA) plus intravitreal bevacizumab (IVB) into patients with center-involving diabetic macular edema (CI-DME).

**Methods:**

In this phase 2/3 randomized controlled pilot trial, sixty-six eyes with CI-DME and best-corrected visual acuity (BCVA) of at most 20/50 Snellen chart were randomly assigned into two groups. Monotherapy arm received sham injection plus 3 monthly IVB doses and combination arm received a single dose of SCTA and 3 monthly IVB doses. The mean improvements in BCVA and Central subfield thickness (CST), over the three-month was considered the main efficacy outcomes.

**Results:**

The mean BCVA improvements were obtained respectively as − 0.20 ± 0.20 log [minimum angle of resolution (MAR)] (*P = 0.004*) and 0.37 ± 0.24 log MAR (*P < 0.001*) in monotherapy and combination arms [between-group analysis (*P = 0.014*)]. Significant improvements were also observed in CST (*P = 0.019*) in the combination arm compared to the other. No adverse events (elevated intraocular pressure, cataract) were observed in any of the study arms.

**Conclusion:**

Significant improvements in BCVA and retinal anatomical outcomes demonstrated the additive effects of SCTA to those of anti-vascular endothelial growth factors with no short-term side effects and this combination appears to be a promising option in the management of patients with CI-DME.

**Trial registration:**

The trial was registered in Iranian Registry of Clinical Trials (IRCT20200314046761N1).

## Background

Diabetes mellitus is estimated to involve over 500 million individuals in upcoming decades. The highly-prevalent diabetic retinopathy should also be considered a threat to vision in human in the near future [1]. Moreover, gaining knowledge about the pathophysiology of diabetic macular edema (DME) as a major cause of visual impairment in patients with diabetic retinopathy [1], can help find effective treatments. Research suggests inflammatory and vascular pathways significantly contribute to macular edema [1, 2]. Numerous studies have addressed anti-inflammatory agents, including intravitreal steroids and also intravitreal methotrexate which suppress many inflammatory cytokines, in treatment-naive patients and those with persistent DME [3–8].

Many studies have investigated the pharmacologic aspects of the suprachoroidal space (SCS) for delivering pharmacological agents, especially triamcinolone acetonide (TA), to the eye [9–14]. The drug concentration achieved in the outer retina and choroid using this route was found to significantly exceed that provided through conventional intravitreal injection. The insignificant drug exposure to anterior segment structures caused by injecting suprachoroidal triamcinolone acetonide (SCTA) was also found to minimize the main adverse effects of steroids, i.e. cataract and elevated intraocular pressure (IOP) [9, 10]. This delivery route therefore appeared to facilitate the treatment of posterior segment disorders.

This manuscript investigated the effect of the novel combination of SCTA and intravitreal bevacizumab (IVB) on managing DME.

## Methods

### Study design

This prospective phase 2/3 triple-blind, uni-center parallel-group randomized controlled pilot trial was conducted to investigate the efficacy of a combination of SCTA and IVB administered in patients with DME. The study designed as per the Declaration of Helsinki was approved by the Ethics Committee of Isfahan University of Medical Sciences, Isfahan, Iran (IR.MUI.MED.REC.1398.410). All the participants were briefed on the benefits/risks of the study, asked to sign informed consent forms and then included in the study. The trial was registered in Iranian Registry of Clinical Trials (IRCT20200314046761N1, first registration date: 21/03/2020).

### Participants

The present study population comprised patients aged at least 18 years with vision loss caused by type 2 diabetes mellitus presenting to the retina clinic of Feiz Eye Hospital affiliated to Isfahan University of Medical Sciences. The eligibility criteria consisted of having center-involving diabetic macular edema (CI-DME) diagnosed with spectral domain optical coherence tomography (SD-OCT) (SECTRALIS, Heidelberg), a central subfield thickness (CST) of at least 320 μm and a baseline best-corrected visual acuity (BCVA) of at most 20/50 Snellen chart, approximately equivalent to 65 Early Treatment Diabetic Retinopathy Study (ETDRS) letter scores [15].

The exclusion criteria encompassed uncontrolled hypertension, history of intravitreal anti-vascular endothelial growth factor (anti-VEGF) injections and focal/grid/pan-retinal laser photocoagulation of at most 3 months in the study eye prior to the first visit, history of intravitreal or peribulbar corticosteroid injections over the previous 6 months and prior suprachoroidal corticosteroid injections or intravitreal corticosteroid implants in the study eye, having the study eye undergo a cataract surgery within the previous 3 months or a significant lens opacity confirmed through expert examinations potentially requiring surgeries within the following 6 months, history of retinal or glaucoma surgery in the study eye, requiring anti-glaucoma drops for lowering IOP, other ocular pathologies in the study eye with potential inadvertent effects on the visual and anatomical outcomes, including corneal haziness and dystrophies, uveitis, retinal and macular dystrophies, active proliferative diabetic retinopathy with or without vitreous hemorrhage, choroidal neovascularization, macular scar and optic disc lesions, systemic conditions contraindicating to using corticosteroids, hypersensitivity to TA or bevacizumab, pregnancy and breastfeeding.

### Sample size and randomization

The sample size was calculated as 30 per group with a test power of 80% and a type I (two-sided) error of 5% corresponding to a mean BCVA difference of 10 ± 15 as our primary outcome reported in ETDRS letter scores for two study arms [16]. Thirty-three eyes were ultimately assigned to each of the study arms considering a drop-out rate of 10%.

Randomized sequences generated using a random number generator in SPSS and permuted blocks of size 4 at a 1:1 ratio were transferred to sealed envelopes, which revealed the next treatment assignment once a participant was included.

### Intervention protocol

**Fig. 1:**
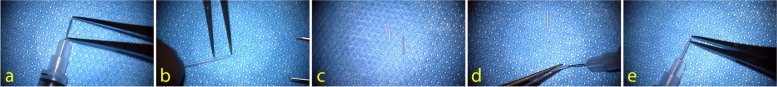
Detailed steps of preparing the custom made needle to perform suprachoroidal triamcinolone acetonide injection. **a** The first step was to measure the entire length of a 30-gauge needle which can penetrate to the eye using the caliper. **b**,** c** The second step was to prepare a plastic sleeve obtained from a branula with the length of 900–1000 μm lower than the needle length, and then autoclaved them. **d**,** e** The plastic sleeves were put on the head of the needle in a way that allowed only 900–1000 μm of the needle to expose and penetrate the eye in order to perform suprachoroidal injection

Every participant received three consecutive anesthetic drops every five minutes before beginning the procedure. During the first injection session, after placing an eyelid speculum and disinfecting the ocular surface with a 10% povidone-iodine solution for 30 seconds in all study eyes, 0.1 ml (4 mg) of TA (Exir Pharmaceutical Co., Iran) was injected into SCS 3–4 mm posterior to the limbus supratemporally with a 900–1000 μm terminally sharp sterile 30-gauge custom made needle in the combination arm. Detailed steps of preparing the needles was shown in Fig. 1.

A different syringe was then used to inject 1.25 mg per 0.05 ml of IVB (Avastin, Roche) into the superior/temporal quadrant 3–4 mm posterior to the limbus. Anterior chamber paracentesis was ultimately performed to reduce IOP. A sham injection performed by pressing the sclera with a needleless syringe was followed by injecting (1.25 mg/0.05 ml) of IVB into the supratemporal quadrant 3–4 mm posterior to the limbus in the monotherapy arm. The patients were monitored in the clinic for about 30 minutes after the injections. Two days later, the injection was performed into the other eye of the patients whose both eyes were included in the study. All the participants received the same dose of IVB per month as that of the first session the following 2 months.

The patients were examined at baseline, and the first injection was performed within 7 days. Follow-ups were performed on the 28-35th day, and 90-100th day of the first injection session. Visual acuity was evaluated with and without refraction using a standard Snellen chart with the distance of 20 ft (6 m). Detailed ophthalmologic examinations of the individual study eyes included slit-lamp biomicroscopy, dilated fundus examination, IOP measurement and SD-OCT, which was used to evaluate CST and macular volume (MV) were then performed. Experts blinded to the grouping and data collected from the previous examinations each performed a single study task.

### Outcomes

The superior efficacy of the combination of SCTA and IVB compared to that of IVB alone, was the primary outcome of this three-months, randomized pilot study which will be achieved through between group analysis of the mean BCVA changes after the study was completed. Secondary outcomes were obtained from comparing the following between the study arms: (1) mean changes in BCVA, 4 weeks after baseline (2) proportion of patients with at least 15 ETDRS letter scores improvement in BCVA after twelve weeks (3) mean changes in CST and MV, four and twelve weeks after baseline (4) proportion of participants requiring additional treatments such as continuing monthly IVB injections, intravitreal corticosteroid injections and focal/grid laser photocoagulation based on expert diagnoses owing to persistent DME after three monthly injections. Persistent DME respectively defined as CST of at least 320 and 305 μm in the males and females was diagnosed through SD-OCT (Heidelberg, Spectralis) [8].

Incidence of acute bacterial endophthalmitis, mean changes in IOP, four and twelve weeks after baseline, incidence of cataract progression and incidence of sub-conjunctival hemorrhage, were considered the possible adverse events.

### Statistical analysis

The continuous and categorical data were reported as mean ± SD and frequency and relative frequency. The normality of the continuous data was evaluated using the Kolmogorov-Smirnov test and Q-Q plots. The non-normal positively-skewed data underwent a logarithmic transform. Continuous, categorical, demographic and clinical characteristics of the study participants were compared between the two groups using the independent t-test and Chi-squared test. Within-group and between-group comparisons were performed in terms of the main outcomes using a linear mixed-effects model. The effects of confounding factors were controlled by adjusting for the baseline outcomes in cases of significant practical or statistical differences. An analyzer blinded to the grouping analyzed the data in SPSS Statistics for Windows, version 16.0 (SPSS Inc., Chicago, Ill., USA).

## Results

### Study participants

**Fig. 2:**
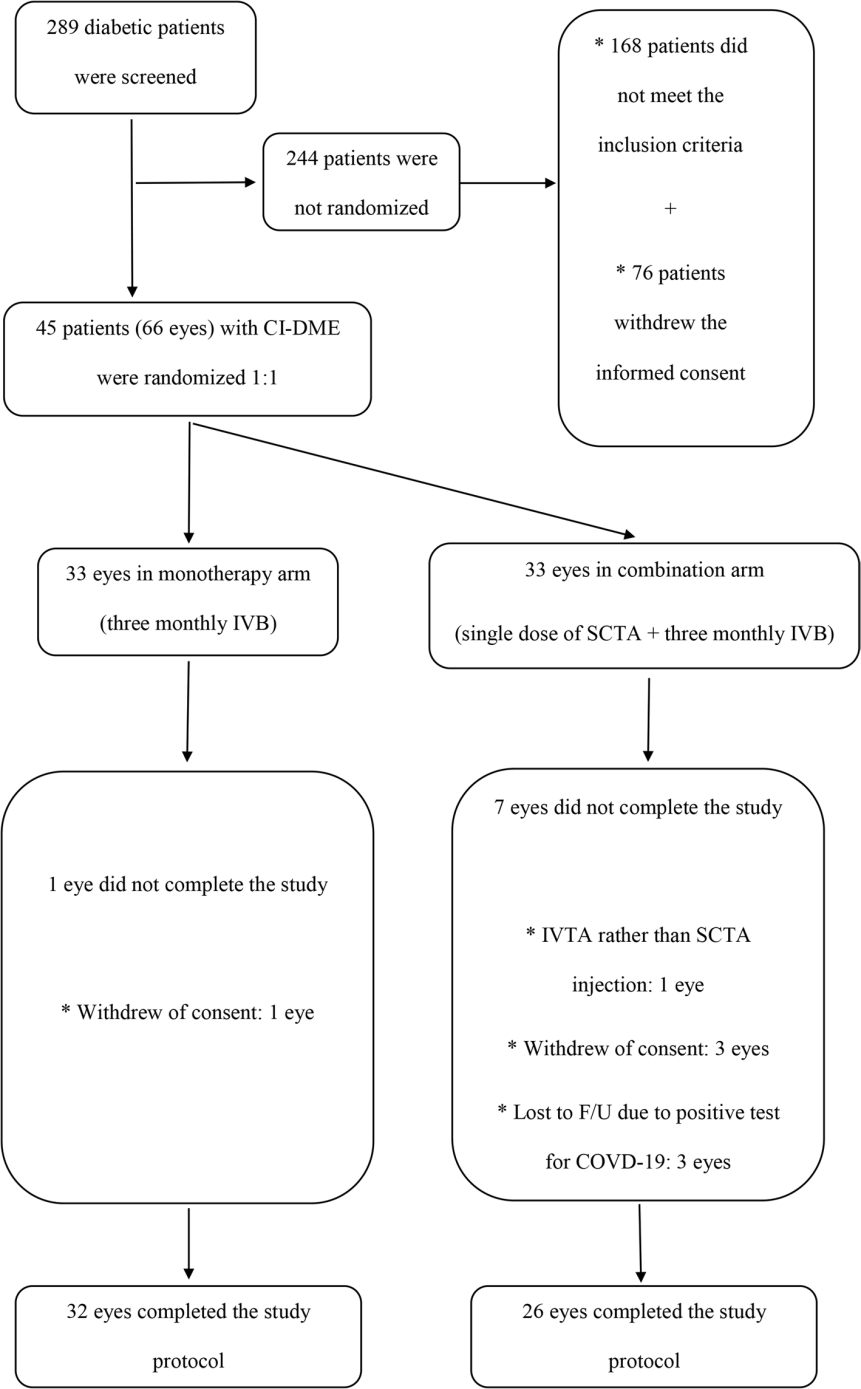
Complete randomization set and patient’s follow-up during the study period. CI-DME: Center involving diabetic macular edema, IVB: Intravitreal bevacizumab, SCTA: Suprachoroidal triamcinolone acetonide, IVTA: Intravitreal triamcinolone acetonide. F/U: follow up

Sixty-six eyes of 45 patients with diabetes and treatment-naive CI-DME primarily recruited based on the eligibility criteria were randomly assigned to the single dose of SCTA + three monthly IVB injections (combination) arm and sham+ three monthly IVB injections (monotherapy) arm. A single eye was excluded from the combination arm owing to intravitreal rather than suprachoroidal injection of TA. Three eyes were excluded from the final analysis in the combination arm due to lost to follow up because of positive COVID-19 test. In addition, four eyes were excluded because of consent withdrawal during the study period. Finally, according to Fig. 2, statistical analyses were performed on 26 eyes in the combination arm and 32 in the monotherapy arm of 39 patients who underwent all the injections and twelve weeks of assessment.

Insignificant differences were observed in demographic and baseline characteristics between the two groups (Table 1). The mean age was 62.8 ± 5.8 years in the monotherapy arm and 62.4 ± 6.2 in the combination arm (*P = 0.8*0). Females accounted for 72% (*n* = 23) of the participants in the monotherapy arm and 62% (*n* = 16) in the combination arm (*P = 0.29*). The mean duration of diabetes was also 13.5 ± 5.7 years in the monotherapy arm and 12.7 ± 5.1 in the combination arm (*P = 0.59*). Moreover, the mean BCVA was 0.79 ± 0.29 log [minimum angle of resolution (MAR)] approximately equivalent to 46 ± 14 ETDRS letter scores in the monotherapy arm and 0.80 ± 0.32 log MAR approximately equivalent to 45 ± 16 ETDRS letter scores in the combination arm at the first visit (*P = 0.91*).

**Table 1:** Baseline demographic and ocular characteristics of the eyes completed the study protocol

Variables	Total *n* = 58	Monotherapy arm (three monthly IVB) *n* = 32	Combination arm (single dose of SCTA + three monthly IVB) *n* = 26	Between group analysis ^a^
**Age, year**				
Mean (SD)	62.6 (5.9)	62.8 (5.8)	62.4 (6.2)	*P* = 0.80
Median (IQR)	63 (50–73)	63 (53–73)	62.5 (50–73)	
**Sex, n (%)**				
Male	19 (32.8%)	9 (28.1%)	10 (38.5%)	*P* = 0.29
Female	39 (67.2%)	23 (71.9%)	16 (61.5%)	
**Diabetes duration, year**				
Mean (SD)	13.2 (5.4)	13.5 (5.7)	12.7 (5.1)	*P* = 0.59
Median (IQR)	12.5 (2–25)	12.5 (2–25)	12.5 (4–22)	
**Previous IVB injection %**				
Yes	27 (46.6%)	15 (46.9%)	12 (46.2%)	*P* = 0.87
No	31 (53.4%)	17 (53.1%)	14 (53.8%)	
**No. of previous IVB injections**				
Mean (SD)	1.8 (2.4)	1.8 (2.4)	1.7 (2.5)	*P* = 0.85
Median (IQR)	0 (0–9)	0 (0–9)	0 (0–9)	
**Lens status**				
Natural (phakic)	41 (70.7%)	23 (71.9%)	18 (69.2%)	*P* = 0.83
Prosthetic IOL	17 (29.3%)	9 (28.1%)	8 (30.8%)	
**Stage of diabetic retinopathy, n (%)**				
Mild to moderate NPDR	9 (15.5%)	4 (12.5%)	5 (19.2%)	
Sever NPDR	45 (77.6%)	26 (81.3%)	19 (73.1%)	*P* = 0.75
Regressed PDR	4 (6.9%)	2 (6.2%)	2 (7.7%)	

^a^ Based on independent samples t-test for numerical variables (age, diabetes duration and number of previous IVB injections) and chi-squared test for categorical variables (sex, previous IVB injection, lens status and stage of diabetic retinopathy)

*Abbreviations*: *IVB* intravitreal bevacizumab, *SCTA* suprachoroidal triamcinolone acetonide, *SD* standard deviation, *IQR* inter-quartile range, *IOL* intraocular lens, *NPDR* non-proliferative diabetic retinopathy, *PDR* proliferative diabetic retinopathy

### Efficacy

#### BCVA

**Fig. 3:**
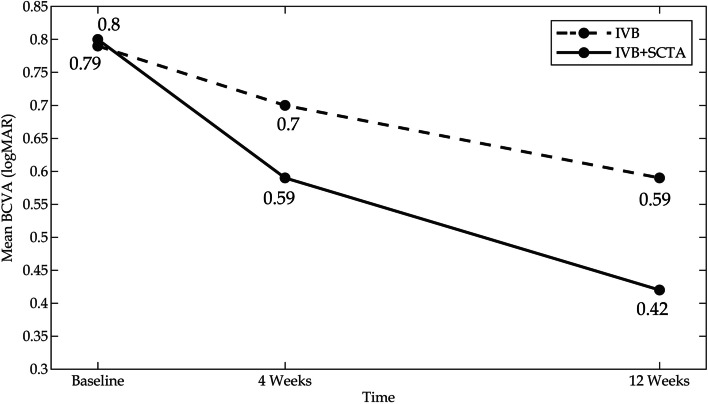
The graph showed mean values of best corrected visual acuity at baseline and the study endpoints in both study arms. BCVA: best corrected visual acuity, Log MAR: logarithm [minimum angle of resolution], IVB: Intravitreal bevacizumab, SCTA: Suprachoroidal triamcinolone acetonide

The mean BCVA was obtained as 0.79 ± 0.29 log MAR at baseline and 0.70 ± 0.27 log MAR 4 weeks later in the monotherapy arm (*P = 0.22*). The two additional IVB injections improved significantly the mean BCVA to 0.59 ± 0.24 log MAR in the monotherapy arm (*P = 0.004*). Adding a single shot of SCTA in the first session significantly improved the mean BCVA from 0.80 ± 0.32 log MAR at baseline to 0.59 ± 0.36 log MAR 4 weeks later (*P = 0.046*) and 0.42 ± 0.30 log MAR, twelve weeks later in the combination arm (*P < 0.001*) (Fig. 3).

The mixed-effects model revealed BCVA improvement was 0.15 log MAR (95% CI, 0.03 to 0.27, *P = 0.014*) superior in the combination arm as compared with the monotherapy arm and helped achieve the primary outcome. Proportion of eyes gaining approximately ≥15 ETDRS letter scores at the final endpoint was also higher in the combination arm but not statistically significant (12 (37.5%) and 12 (46.2%) in the monotherapy and combination arms respectively, *P = 0.59*) (Table 2).

**Table 2:** Outcomes at 3-months follow up

Outcome	Monotherapy arm (three monthly IVB) *n* = 32	Combination arm (single dose of SCTA + three monthly IVB) *n* = 26	Mean difference (combination – monotherapy) (95% CI) ^a^	Linear mixed-effects model analysis (combination vs monotherapy) (95% CI) ^b^
**Visual acuity (log MAR)**	–			
Baseline ^c^	0.79 (0.29)	0.80 (0.32)	0.00 (− 0.15 to 0.17) *P = 0.91*	–
4 weeks ^c^	0.70 (0.27)	0.59 (0.36)	–	–
Change (4 weeks – baseline) ^d^	- 0.09 (0.07)	- 0.20 (0.10)	- 0.11 (− 0.21 to - 0.01) *P = 0.010*	–
Within group analysis ^e^	*P = 0.22*	*P = 0.046*	–	–
12 weeks ^c^	0.59 (0.24)	0.42 (0.30)	–	–
Change (12 weeks – baseline) ^d^	- 0.20 (0.07)	- 0.37 (0.09)	- 0.17 (− 0.38 to - 0.05) *P = 0.004*	- 0.15 (− 0.27 to - 0.03) *P = 0.014*
Within group analysis ^e^	*P = 0.004*	*P < 0.001*		–
**Visual acuity (ETDRS Letter scores)**	–			
Baseline	46 (14)	45 (16)	0 (− 8 to 8) *P = 0.91*	–
4 weeks	50 (13)	55 (18)	–	–
Change (4 weeks – baseline)	4 (4)	10 (5)	6 (1 to 11) *P = 0.010*	–
Within group analysis	*P = 0.22*	*P = 0.046*	–	–
12 weeks	55 (12)	64 (15)	–	–
Change (12 weeks – baseline)	10 (3)	18 (4)	9 (3 to 14) *P = 0.005*	7 (1 to 13) *P = 0.018*
Within group analysis	*P = 0.004*	*P < 0.001*	–	–
No.(%) ≥ 15 ETDRS Letter scores improvement	12 (37.5%)	12 (46.2%)	*P = 0.59* ^f^	*–*
**Central subfield thickness** **(μm)**	–			
Baseline	520 (116)	524 (153)	4 (− 67 to 75) *P = 0.91*	–
4 weeks	444 (117)	337 (107)	–	–
Change (4 weeks – baseline)	- 76 (31)	- 187 (42)	- 111 (− 270 to - 48) *P = 0.010*	–
Within group analysis	*P = 0.015*	*P < 0.001*	–	–
12 weeks	413 (109)	348 (132)	–	–
Change (12 weeks – baseline)	- 108 (28)	- 176 (37)	- 69 (− 131 to - 7) *P = 0.038*	- 48 (− 89 to - 8) *P = 0.019*
Within group analysis	*P < 0.001*	*P < 0.001*	–	–
No. below sex-specific normal threshold values (%) ^g^	8 (25%)	14 (53.8%)	*P = 0.032* ^f^	*–*
**Macular volume (mm**^**3**^**)**	–			
Baseline	11.4 (1.8)	11.3 (2.3)	- 0.1 (− 1.2 to 1) *P = 0.83*	–
4 weeks	10.7 (1.5)	9.6 (1.1)	–	–
Change (4 weeks – baseline)	- 0.7 (0.4)	- 1.6 (0.6)	- 0.9 (− 2.1 to - 0.3) *P = 0.027*	–
Within group analysis	*P = 0.11*	*P = 0.007*	–	–
12 weeks	10.4 (1.5)	9.9 (1.9)	–	–
Change (12 weeks – baseline)	- 1.0 (0.4)	- 1.4 (0.5)	- 0.4 (−1.0 to 0.1) *P = 0.19*	- 0.5 (− 1.0 to - 0.1) *P = 0.027*
Within group analysis	P = 0.020	*P = 0.011*	–	–
**Intraocular pressure (mmHg)**	–			
Baseline	14.9 (2.7)	15.3 (3.2)	0.4 (− 1.2 to 1.9) *P* = 0.65	–
4 weeks	15.0 (1.9)	16 (3.1)	–	–
Change (4 weeks – baseline)	0.1 (0.6)	0.7 (0.9)	0.6 (− 0.2 to 1.5) *P* = 0.18	–
Within group analysis	*P = 0.93*	*P = 0.42*	–	
12 weeks	15.5 (2.2)	16.1 (2.6)	–	
Change (12 weeks – baseline)	0.6 (0.6)	0.8 (0.8)	0.2 (− 0.8 to 1.2) *P = 0.65*	0.6 (− 0.2 to 1.4) *P = 0.13*
Within group analysis	*P = 0.28*	*P = 0.31*	–	–

^a^ Comparing the mean differences of each study outcome was performed using independent samples t-test

^b^ Adjustment was made for baseline values of study outcomes when a practical or statistical significance was detected

^c^ Values of each study outcome are reported as mean (SD) at baseline, four-weeks and twelve-weeks measurements

^d^ Values of changes (4 weeks – baseline, 12 weeks – baseline) are reported as mean (SEM)

^e^ Within group analysis of each study outcome was performed using linear mixed-effects model

^f^ Based on chi-squared test

^g^ Sex-specific normal threshold values were defined as central subfield thickness less than 320 μm in male and 305 μm in female participants measured by spectral domain optical coherence tomography (Heidelberg, Spectralis) [8]

*Abbreviations*: *IVB* intravitreal bevacizumab, *SCTA* suprachoroidal triamcinolone acetonide, *CI* confidence interval, *Log MAR* logarithm [minimum angle of resolution], mean, *ETDRS* Early Treatment Diabetic Retinopathy Study, *SD* standard deviation, *SEM* standard error of mean, *ETDRS* early treatment diabetic retinopathy study

#### Retinal thickness and volume

**Fig. 4:**
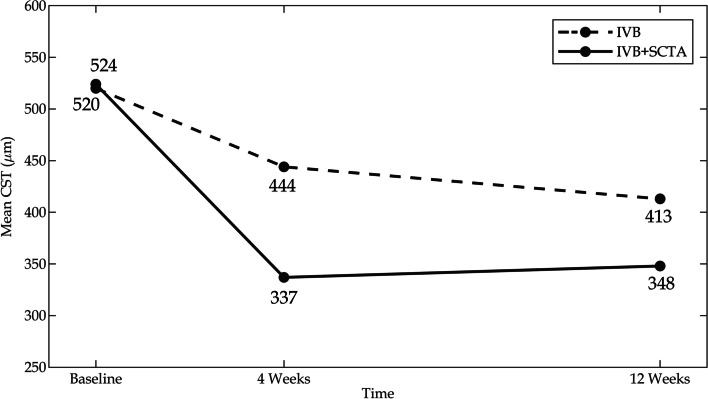
The graph showed mean values of central subfield thickness at baseline and the study endpoints in both study arms. CST: central subfield thickness, IVB: Intravitreal bevacizumab, SCTA: Suprachoroidal triamcinolone acetonide

Significant reductions were observed in CST between baseline and the study endpoints in the monotherapy arm. The mean decrease in CST was 76 μm (95% CI 15 to 138, *P = 0.015*) after 4 weeks and 108 μm (95% CI 51 to 164, *P < 0.001*) after twelve weeks. The mean CST also significantly decreased in the combination arm from 524 ± 153 μm at baseline to 337 ± 107 μm (*P < 0.001*) after 4 weeks and 348 ± 132 μm (*P < 0.001*) after completing the study (Fig. 4). Moreover, between-group analysis revealed significant reductions in retinal thickness in the combination arm. According to the mixed-effects model, adding a single dose of SCTA caused a 48-μm reduction in CST in the combination arm compared to that in the monotherapy arm (95% CI 8 to 89, *P = 0.019*) (Table 2).

On the 12th week, a normal CST was observed based on its gender-specific normal threshold values in 8 (25%) and 14 (53.8%) eyes in the monotherapy and combination arms, respectively. The statistical analyses revealed the synergistic effects of a single dose of SCTA, which significantly increased the normal CST values with an odds ratio of 3.5 (95% CI 1.2 to 10.6, *P = 0.032*).

**Fig. 5:**
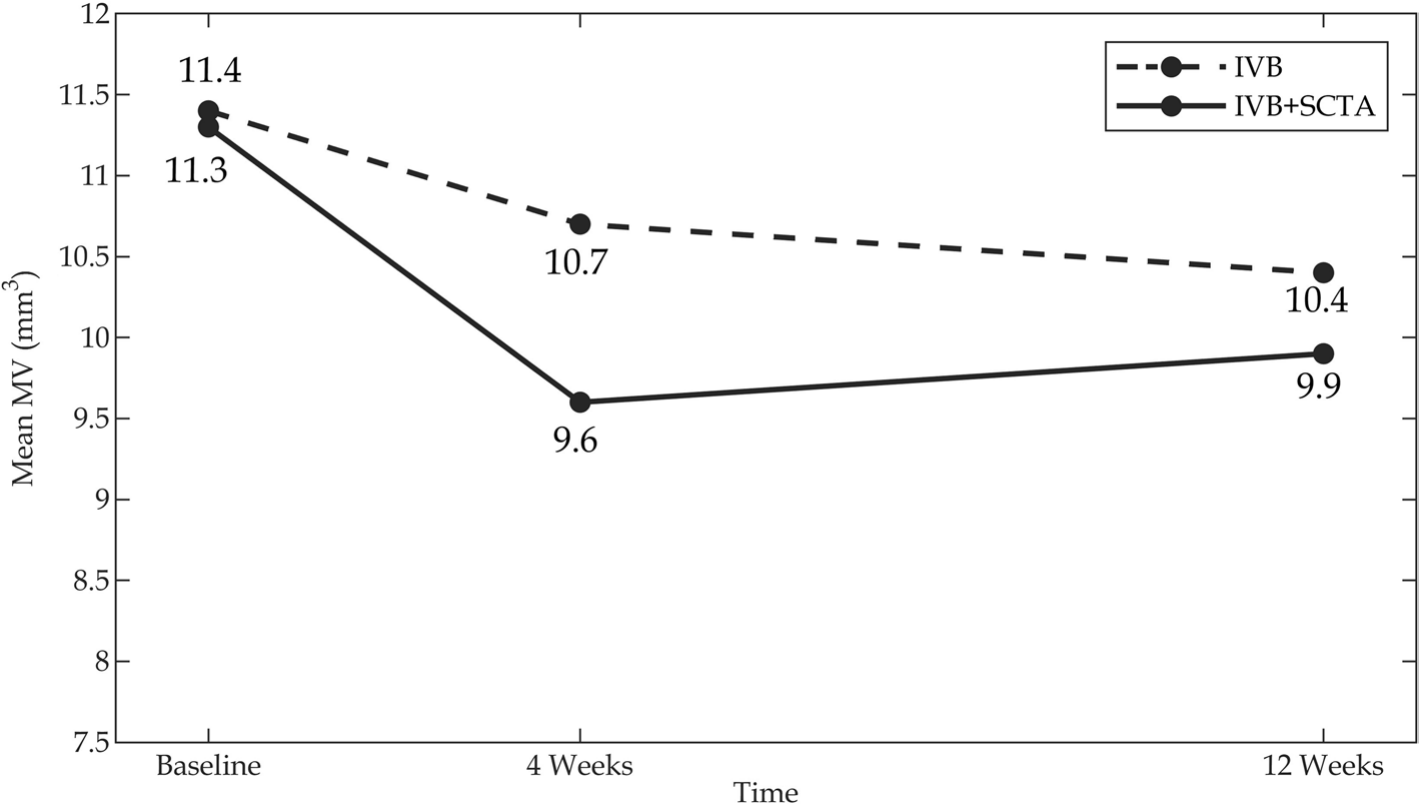
The graph showed mean values of macular volume at baseline and the study endpoints in both study arms. MV: macular volume, IVB: Intravitreal bevacizumab, SCTA: Suprachoroidal triamcinolone acetonide

In addition to CST, MV significantly decreased in both study arms. After four and twelve weeks, the mean resolution in MV was respectively obtained as 0.7mm^3^ (95% CI − 0.2 to 1.6, *P = 0.11*) and 1.0 mm^3^ (95% CI 0.2 to 1.8, *P = 0.020*) in the monotherapy arm and as 1.6mm^3^ (95% CI 0.5 to 2.8, *P = 0.007*) and 1.4 mm^3^ (95% CI 0.3 to 2.4, *P = 0.011*) in the combination arm (Fig. 5). Between-group comparisons using mixed-effects model revealed the significantly higher MV resolution in the combination arm (0.5 mm^3^, 95% CI 01 to 1.0, *P = 0.027*) (Table 2).

#### Adverse events

Acute endophthalmitis was observed in none of the study eyes. TA was inadvertently injected into the vitreous cavity of 2.2% (*n* = 1) of the eyes in the combination arm, and the case was excluded from the final analysis.

There was no significant IOP rising which needed medical or surgical treatment (over 10, 25 or 30 mmHg) observed in any of the participants. The mean IOP respectively obtained as 14.9 ± 2.7, 15 ± 1.9 and 15.5 ± 2.2 mmHg at baseline and after four and twelve weeks in the monotherapy arm and as 15.3 ± 3.2, 16 ± 3.1 and 16.1 ± 2.6 mmHg in the combination arm. There was no significant IOP rising noticed in each groups during the study period (Table 2). Cataract was observed in neither of the groups. Sub-conjunctival hemorrhage respectively observed in 65.4 and 43.8% of the eyes in the combination and monotherapy arms (*P = 0.006*) self-healed within 2 weeks after the injections in all the participants and caused no complications.

## Discussion

This two-arm randomized pilot trial was conducted to evaluate the efficiency of adding a single dose of SCTA to IVB, as a popular anti-VEGF agent especially in developing countries, in managing CI-DME in treatment-naive eyes. The findings showed significant clinical and statistical improvements in BCVA and retinal anatomical outcomes in the eyes injected with this novel combination compared to in those receiving IVB.

Today, anti-VEGF agents are commonly used as the first-line therapy for macular edema caused by retinal diseases such as age-related macular degeneration, retinal vein occlusion, certain retinal tumors and diabetic retinopathy [17–20]. Despite their global applications and undeniable benefits, these agents have been reported to cause numerous adverse ocular and systemic events and different responses in patients [17, 18, 21]. Bressler et al. found six monthly injections of ranibizumab to insignificantly resolve macular edema in approximately 40% of the eyes. They reported chronic DME in 55.8 and 40.1% of the patients, respectively, despite continuing the injections based a specific two- and three-year follow-up protocol. Furthermore, they found macular edema to be more likely to persist in the eyes managed with IVB injections (72.9% after twelve weeks, 65.6% after 24 weeks and 68.2% after 2 years) [22]. Similarly, the present findings suggested CST did not fall below the gender-specific normal thresholds in 75% of the participants managed with three consecutive monthly IVB injections.

Reviewing the pathophysiology of DME can clarify the high unresponsiveness rates observed. Das et al. explained the pathophysiology of diabetic retinopathy by reporting four major biochemical mechanisms induced by hyperglycemia [1]. These pathways upregulate many mediators, including VEGFs and inflammatory cytokines such as tumor necrosis factor, interleukin 6, interleukin 8, intercellular adhesion molecule-1 and matrix metalloproteinases, and ultimately cause blood-retinal barrier breakdown [1, 2]. Kim et al. predicted the response rates to IVB and intravitreal triamcinolone acetonide (IVTA) injections by reporting that these mechanisms can cause different OCT patterns. They observed the best responses to IVB in cases with the sponge-like patterns of diffuse retinal thickening mainly caused by increased vascular permeability. More effective responses to intravitreal steroids were also reported in cystoid patterns mainly caused by Müller cell death and its liquefaction [21]. Given that anti-VEGF agents cannot entirely inhibit the underlying mechanisms of DME, other medical modalities with anti-inflammatory mechanisms are required for obtaining better results.

Compared to IVB alone, IVB in combination with fusadil hydrochloride as a Rho/Rho kinase inhibitor was found by Ahmadieh et al. to cause better and prolonged visual outcomes and no significant adverse effects on patients with CI-DME [16].

The properties of corticosteroids have been well addressed in literature. ^5–7,23^ The one- and two-year results of BEVORDEX trial showed approximately equal improvements in BCVA and better anatomical outcomes in patients receiving intravitreal dexamethasone implant compared to in those injected with IVB [6, 7]. Maturi et al. found adding intravitreal dexamethasone to monthly ranibizumab injections ineffective in improving visual outcomes in patients with persistent DME [8]. Significant complications reported after administering corticosteroids included elevated IOP and cataract [10, 23]. Intravitreal corticosteroids and implants were therefore considered a second-line therapy for DME.

SCS lies between the choroid and sclera in healthy eyes. Exploring the safety and pharmacodynamics of SCTA injections in rabbit models, Chen et al. reported a significantly-higher drug concentration in the posterior retina than in the anterior segment. They also found that the resolution of both anterior and posterior segment inflammations was also significantly higher in suprachoroidal drug delivery compared to in posterior sub-tenon injection of TA [9]. According to Habot-Wilner et al. injecting pharmacological agents into SCS precipitates the distribution to the posterior segment. They also reported the TA concentration to be twelve times higher in the posterior segment and only 5% in aqueous humor, ciliary body and lens compared to IVTA injections [10]. Given its negligible concentration in anterior segment structures, SCTA injection appears to significantly decrease the risk of elevated IOP and cataract compared to intravitreal drug delivery [9]. Major adverse events such as elevated intraocular pressure and clinically-significant cataract were not observed in any of the present study participants.

According to the Tanzanite clinical trial, SCTA plus intravitreal aflibercept (IVA) was well tolerated compared to IVA alone in patients with retinal vein occlusion. The number of injections also significantly decreased and BCVA and retinal thickness significantly improved in the combination arm [14]. Yeh et al. investigated the effects of two doses of SCTA injections with a three-month interval on macular edema caused by noninfectious uveitis. They reported significant improvements in BCVA and resolution of edema. They also found the injection not to cause remarkable adverse ocular events [12]. Wykoff et al. administered either a single or multiple SCTA injections combined with a single IVA injection in patients with naive or persistent DME, and found the CST resolution to exceed 50% in almost 90% of the patients after 6 months [13]. In line with these studies, we obtained significant improvements in anatomical and functional outcomes through adding SCTA injections.

The present study limitations mainly comprised its small sample and relatively short-term follow-ups. Given the COVID-19 pandemic in Iran, the eight-week follow-up was not completed in the majority of the participants, resulting in unreliable data. Lack of a group with the combination injection of posterior subtenon triamcinolone acetonide and IVB was another limitation of this study.

To the best of the authors’ knowledge, this study pioneered the investigation of the synergistic effects of SCTA and IVB in patients with diabetes. The strengths of the present research encompassed its randomization, the patients and evaluators masking to avoid possible bias, adherence to the study protocol and not depriving the patients from a confirmed treatment (ant-VEGFs). It is recommended that large clinical trials with longer-term follow-ups be performed to investigate the synergistic and sole effects of SCTA injections and report its potential adverse effects in patients with DME.

## Data Availability

The datasets used and/or analyzed during the current study are available from the corresponding author on reasonable request.

## Abbreviations

DME: Diabetic macular edema
SCS: Suprachoroidal space
TA: Triamcinolone acetonide
SCTA: Suprachoroidal triamcinolone acetonide
IOP: Intraocular pressure
IVB: Intravitreal bevacizumab
CI-DME: Center-involved diabetic macular edema
SD-OCT: Spectral domain optical coherence tomography
CST: Central subfield thickness
BCVA: Best corrected visual acuity
ETDRS: Early treatment diabetic retinopathy study
anti-VEGF: anti-vascular endothelial growth factor
MV: Macular volume
IVTA: Intravitreal triamcinolone acetonide
IVA: Intravitreal aflibercept
